# Ureteric valve: Case report with an insight into anatomy, embryology, presentation and management

**DOI:** 10.4103/0970-1591.44269

**Published:** 2008

**Authors:** Rahul K. Gupta, S. S. Borwankar, Sandesh V. Parelkar

**Affiliations:** Department of Pediatric Surgery, King Edward Memorial Hospital, Mumbai - 400 012, Maharashtra, India

**Keywords:** Ureteric stricture, ureteric valve

## Abstract

Congenital ureteric obstruction caused by a ureteric valve is an exceedingly rare entity. Our patient, a nine-year-old male, had undergone evaluation for recurrent pain in the abdomen and was diagnosed as a case of left hydronephrosis on ultrasound abdomen. Intravenous urography and magnetic resonance urography showed incomplete duplex system on the right side along with left hydronephrosis and hydroureter. Cystoscopy with left ascending gram followed by excision of lower third of ureter along with valve and Cohen's ureteroneocystostomy was done. Histopathology revealed Type II ureteric valve. A high index of suspicion is required to make a correct preoperative diagnosis.

## INTRODUCTION

Congenital ureteric valves are very rare. Children with this disorder are often misdiagnosed as having either ureterovesical junction obstruction or primary megaureter. We report a case of congenital ureteric valve with a review of the literature.

## CASE REPORT

**Figure 1:**
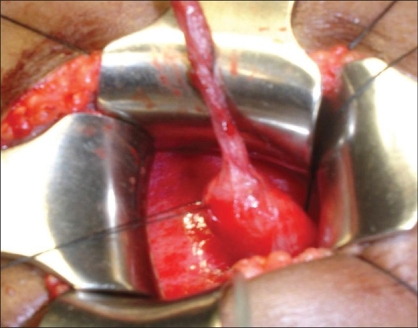
Narrowed zone at junction of dilated upper two-thirds and narrowed lower one-third of ureter

**Figure 2:**
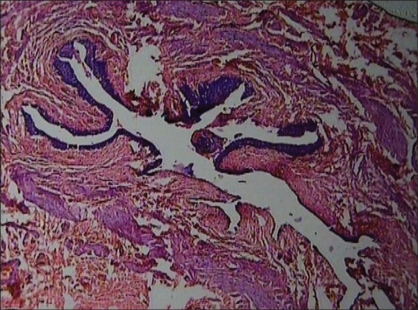
Histopathology showing Type II ureteric valve

A nine-year-old male child presented with recurrent abdominal pain in the left lumbar region since four years. A renal ultrasound revealed left hydronephrosis. Voiding cystourethrogram was normal .Renal scan showed a normally functioning left kidney with split renal function of 37% and an obstructive pattern. The right kidney was normal. Intravenous urogram and magnetic resonance urography showed incomplete duplication of the right side with left hydronephrosis and hydroureter. During cystoscopy, a 3 Fr ureteric catheter could not be passed up to the renal pelvis, and was obstructed at a distance of 4-5 cm from the vesicoureteric junction. Dye injected through the catheter did not delineate the upper two-thirds of the ureter. Patient was posted for Cohen's ureteroneocystostomy. On intravesical mobilization of the ureter, an obvious narrowing was seen along the ureter at a distance of 4-5 cm from the bladder wall with proximal dilatation of the upper two-thirds of the ureter [Figure 1]. An incision was made over the narrowing, which revealed a ureteric valve with an eccentric pinpoint orifice. Lower third of the ureter along with the valve was excised and Cohen's ureteroneocystostomy was done. Pathological examination revealed Type II ureteric valve lined by transitional epithelium overlying a stroma containing smooth muscle fibers and fibrous tissue [Figure 2]. Patient had uneventful recovery. On one-year follow-up renal scan, there is 47% split renal function of left kidney without obstructive pattern.

## DISCUSSION

Congenital ureteric valves are a rare cause of ureteric obstruction. Only 60 cases of ureteric valves have been reported since Wolfler described his first case in 1877.[1]

For diagnosis of ‘ureteric valves’, the following criteria should be present: (1) Presence of transverse folds of the ureteric mucosa containing bundles of smooth muscle fiber on histologic examination, (2) signs of obstructive disease above the valve with a normal ureter below it, and (3) no other evidence of mechanical or functional obstruction.[2]

According to Rabinowitz,[3] ureteric valves can be classified as Type I or Type II, with Type I having smooth muscle present within the leaflet and Type II having smooth muscle at the base only.

On the basis of previously reported cases ureteric valves can be classified morphologically into both cusp-like (leaflet) and diaphragmatic or annular type.[3] Our case is a cusp-like (leaflet) Type II true ureteric valve, as demonstrated by histopathology.

The embryogenesis of ureteric valves remains unclear. Two major theories presently exist: the persistence of Chwalle's membrane and physiological folds. The persistence of Chwalle's membrane might explain the presence of lower ureteric valves.[2] Chwalle's membrane is an epithelial membrane in the lower portion of the ureteric lumen and is a normal feature of ureteric development at six weeks of gestation. During the eighth week of gestation the membrane ruptures under the pressure of urine excretion. Partial rupture of the membrane may result in a retained membrane that would constitute a ureteric valve. However, this theory does not explain multiple valves in one ureter or valves in the upper or mid-ureter.[4]

At the fifth week of embryologic life, the ureteric bud originating from the mesonephric (Wolfian) duct grows towards the metanephric blastema. On the other hand, the metanephros undergoes an ascent towards its definitive location. If the ureteric growth is faster than the renal migration, then a “ureteric fold” would form. Ureteric valves are the persistence of exaggerated and obstructive fetal physiologic ureteric folds. This theory seems more compatible with multiple valves in one ureter.[4] The distribution of valves within the ureter is reported as 50% in the proximal ureter, 17% in mid-ureter, and 33% in the distal ureter. One case of multiple valves on the same side has also been described.[1] Bilateral involvement is exceedingly rare, and has been described only by Wall and Wachter in 1952[2] and Paul Daher in 2007.[1]

A patient of ureteric valve can present as a lump in the abdomen due to hydronephrosis, abdominal pain or can be detected incidentally while evaluating suspected cases of megaureters, ureteropelvic or ureterovesical junction obstruction.

More than 50% of cases with ureteric valves also have associated urinary anomalies, including ureteric duplication, reflux, ectopic ureter, and contralateral hypoplastic kidney or renal agenesis.[3] Our patient had incomplete duplex system on the right side.

The differential diagnosis is congenital ureteric stricture, which is rare, usually found in early adulthood, but often revealed by hydronephrosis on antenatal ultrasound.[1] The stricture corresponds to segmental ureteric fibrosis, usually associated with smooth muscle hypoplasia. The ureteric lumen is usually narrow but sometimes can be of normal caliber. The stricture can be present anywhere in the ureter.[1]

Treatment of ureteric valve depends on its location and the severity of renal damage and includes pyeloureterostomy, primary ureteroureterostomy, or excision of leaflet and ureteric reimplantation in cases of distal involvement.[3] Endoscopic incision is also thought to be a useful treatment for the ureteric valves.[1] Recently, antenatal ablation of ureteric valve by Nd-YAG laser has also been reported.[5]

In our case, on the basis of all radiological investigations, the initial provisional diagnosis was left ureterovesical junction obstruction. Despite the availability of advanced diagnostic radiological modalities, a high index of suspicion is needed to make the diagnosis of ureteric valve as a cause of unilateral hydronephrosis and hydroureter. The key message is that diagnosis of ureteric valve should be kept in mind and needs to be confirmed by cystoscopy followed by retrograde ureterography, while evaluating a case of hydronephrosis and hydroureter.
